# 4-Iodo­anilinium nitrate

**DOI:** 10.1107/S1600536810016740

**Published:** 2010-05-12

**Authors:** Xue-qun Fu

**Affiliations:** a Ordered Matter Science Research Center, Southeast University, Nanjing 210096, People’s Republic of China

## Abstract

In the title compound, C_6_H_7_IN^+^·NO_3_^−^, π–π stacking inter­actions [centroid–centroid distances = 4.014 (4) and 4.029 (4) Å]  stabilize the crystal structure and strong N—H⋯O and N—H⋯N hydrogen bonds link the cations and anions into zigzag chains running parallel to the *c* axis. The asymmetric unit contains two unique cations and anions

## Related literature

For background to phase-transition materials, see: Li *et al.* (2008 ▶); Zhang *et al.* (2009 ▶).
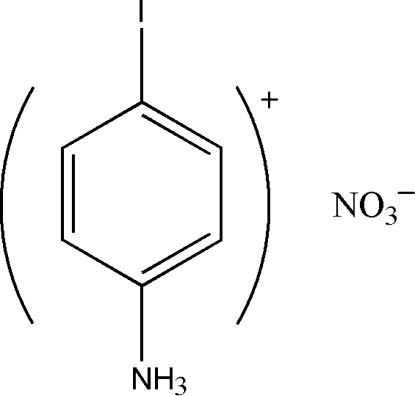


## Experimental

### 

#### Crystal data

C_6_H_7_IN^+^·NO_3_^−^
*M* *_r_* = 282.04
Monoclinic, 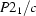

*a* = 21.847 (4) Å
*b* = 5.6103 (11) Å
*c* = 15.928 (3) Å
β = 110.11 (3)°
*V* = 1833.2 (6) Å^3^
*Z* = 8
Mo *K*α radiation
μ = 3.47 mm^−1^
*T* = 298 K
0.40 × 0.30 × 0.20 mm

#### Data collection

Rigaku SCXmini diffractometer
Absorption correction: multi-scan (*CrystalClear*; Rigaku, 2005 ▶) *T*_min_ = 0.5, *T*_max_ = 0.5
17732 measured reflections
4196 independent reflections
3075 reflections with *I* > 2σ(*I*)
*R*_int_ = 0.041

#### Refinement

*R*[*F* ^2^ > 2σ(*F* ^2^)] = 0.056
*wR*(*F* ^2^) = 0.112
*S* = 1.13
4196 reflections
217 parameters
H-atom parameters constrained
Δρ_max_ = 1.07 e Å^−3^
Δρ_min_ = −0.78 e Å^−3^

### 

Data collection: *CrystalClear* (Rigaku, 2005 ▶); cell refinement: *CrystalClear*; data reduction: *CrystalClear*; program(s) used to solve structure: *SHELXS97* (Sheldrick, 2008 ▶); program(s) used to refine structure: *SHELXL97* (Sheldrick, 2008 ▶); molecular graphics: *SHELXTL* (Sheldrick, 2008 ▶); software used to prepare material for publication: *PRPKAPPA* (Ferguson, 1999 ▶).

## Supplementary Material

Crystal structure: contains datablocks I, global. DOI: 10.1107/S1600536810016740/jh2153sup1.cif

Structure factors: contains datablocks I. DOI: 10.1107/S1600536810016740/jh2153Isup2.hkl

Additional supplementary materials:  crystallographic information; 3D view; checkCIF report

## Figures and Tables

**Table 1:** Hydrogen-bond geometry (Å, °)

*D*—H⋯*A*	*D*—H	H⋯*A*	*D*⋯*A*	*D*—H⋯*A*
N1—H1*B*⋯O4^i^	0.89	2.18	2.945 (8)	144
N1—H1*C*⋯O2^ii^	0.89	2.24	2.819 (8)	122
N1—H1*C*⋯O5^i^	0.89	2.46	3.010 (8)	120
N1—H1*D*⋯O3^i^	0.89	2.46	2.898 (8)	111
N1—H1*D*⋯I1^ii^	0.89	3.15	3.990 (6)	157
N2—H2*B*⋯O6^iii^	0.89	2.03	2.892 (8)	162
N2—H2*B*⋯O4^iii^	0.89	2.44	3.104 (8)	132
N2—H2*B*⋯N4^iii^	0.89	2.55	3.372 (8)	153
N2—H2*C*⋯O3^iv^	0.89	1.91	2.795 (7)	173
N2—H2*C*⋯N3^iv^	0.89	2.56	3.407 (8)	159
N2—H2*C*⋯O1^iv^	0.89	2.59	3.264 (8)	133
N2—H2*D*⋯O5^v^	0.89	2.18	3.018 (8)	157
N2—H2*D*⋯O6^v^	0.89	2.28	3.053 (8)	145
N2—H2*D*⋯N4^v^	0.89	2.58	3.459 (9)	170

Symmetry codes: (i) 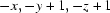; (ii) 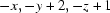; (iii) 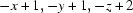; (iv) 
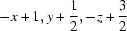
; (v) 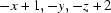.

**Fig. 1.**
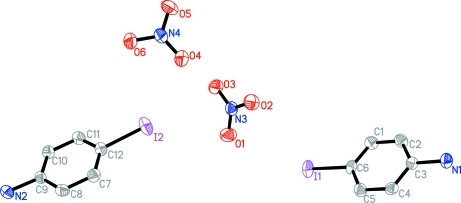
The molecular structure of the title compound, with the atomic numbering scheme. Displacement ellipsoids are drawn at the 30% probability level, and all H atoms have been omitted for clarity.

**Fig. 2.**
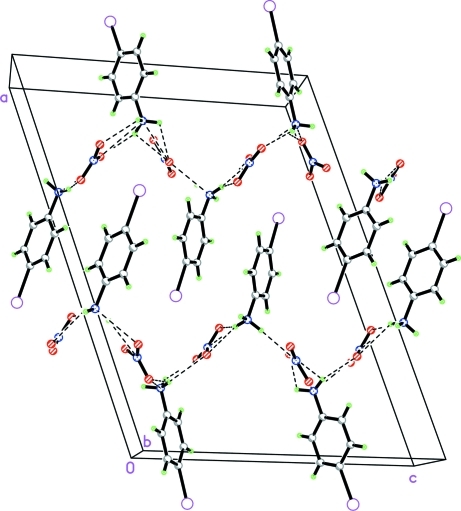
A view of the packing of the title compound, stacking along the c axis. Dashed lines indicate hydrogen bonds.

## supplementary crystallographic information

### Comment

As a continuation of our study of phase transition materials, including organic ligands (Li *et al.*, 2008), metal-organic coordination compounds (Zhang *et al.*, 2009 ), organic-inorganic hybrids, we studied the dielectric properties of the title compound, unfortunately, there was no distinct anomaly observed from 93 K to 380 K, (m.p. 408 K-410 K). In this article, the crystal structure of (I) has been presented.

The asymmetric unit of the title compound is built up from two 4-iodobenzenammnium cations wherein the dihedral angle between plans formed by non-hydrogen atoms is 15.3 (2)°,and two nitrate radical anions (Fig.1). The π-π packing interaction of adjacent benzene rings with Cg(1)—Cg(1), 4.029 (4)Å; Cg(2)—Cg(2), 4.014 (4)Å [Cg(1) and Cg(2) are the centroids of benzene rings, where Cg(1): C(1) to C(6); Cg(2): C(7) to C(12)], make great contribution to the stability of the crystal structure. The strong intermolecular N—H···O (N···O distances 2.795 (7)-3.264 (8)Å) and N—H···N (N···N distances 3.372 (8)-3.459 (9)Å) hydrogen bonding link cations and anions into zigzag chains along *c *axis.

### Experimental

Single crystals of 4-iodoanilinium nitrate were prepared by slow evaporation at room temperature of an ethanol solution of equal molar 4-iodobenzenamine and nitrate acid.

### Refinement

Positional parameters of all the H atoms were calculated geometrically and were allowed to ride on the C and N atoms to which they are bonded, with *U*_iso_(H) = 1.2U_eq_(C),

*U*_iso_(H) = 1.2U_eq_(N).

### Crystal data

**Table:**

C_6_H_7_IN^+^·NO_3_^−^	*F*(000) = 1072
*M**_r_* = 282.04	*D*_x_ = 2.044 Mg m^−^^3^
Monoclinic, *P*2_1_/*c*	Mo *K*α radiation, λ = 0.71073 Å
Hall symbol: -P 2ybc	Cell parameters from 7323 reflections
*a* = 21.847 (4) Å	θ = 3.2–27.6°
*b* = 5.6103 (11) Å	µ = 3.47 mm^−^^1^
*c* = 15.928 (3) Å	*T* = 298 K
β = 110.11 (3)°	Prism, colourless
*V* = 1833.2 (6) Å^3^	0.40 × 0.30 × 0.20 mm
*Z* = 8	

### Data collection

**Table:**

Rigaku SCXmini diffractometer	4196 independent reflections
Radiation source: fine-focus sealed tube	3075 reflections with *I* > 2σ(*I*)
graphite	*R*_int_ = 0.041
Detector resolution: 13.6612 pixels mm^-1^	θ_max_ = 27.5°, θ_min_ = 3.2°
ω scans	*h* = −28→28
Absorption correction: multi-scan (*CrystalClear*; Rigaku, 2005)	*k* = −7→7
*T*_min_ = 0.5, *T*_max_ = 0.5	*l* = −20→20
17732 measured reflections	

### Refinement

**Table:**

Refinement on *F*^2^	Primary atom site location: structure-invariant direct methods
Least-squares matrix: full	Secondary atom site location: difference Fourier map
*R*[*F*^2^ > 2σ(*F*^2^)] = 0.056	Hydrogen site location: inferred from neighbouring sites
*wR*(*F*^2^) = 0.112	H-atom parameters constrained
*S* = 1.13	*w* = 1/[σ^2^(*F*_o_^2^) + (0.0174*P*)^2^ + 9.5262*P*] where *P* = (*F*_o_^2^ + 2*F*_c_^2^)/3
4196 reflections	(Δ/σ)_max_ < 0.001
217 parameters	Δρ_max_ = 1.07 e Å^−^^3^
0 restraints	Δρ_min_ = −0.78 e Å^−^^3^

### Special details

**Table:**

Geometry. All esds (except the esd in the dihedral angle between two l.s. planes) are estimated using the full covariance matrix. The cell esds are taken into account individually in the estimation of esds in distances, angles and torsion angles; correlations between esds in cell parameters are only used when they are defined by crystal symmetry. An approximate (isotropic) treatment of cell esds is used for estimating esds involving l.s. planes.
Refinement. Refinement of *F*^2^ against ALL reflections. The weighted *R*-factor wR and goodness of fit *S* are based on *F*^2^, conventional *R*-factors *R* are based on *F*, with *F* set to zero for negative *F*^2^. The threshold expression of *F*^2^ > σ(*F*^2^) is used only for calculating *R*-factors(gt) etc. and is not relevant to the choice of reflections for refinement. *R*-factors based on *F*^2^ are statistically about twice as large as those based on *F*, and *R*- factors based on ALL data will be even larger.

### Fractional atomic coordinates and isotropic or equivalent isotropic displacement parameters (Å^2^)

**Table:**

	*x*	*y*	*z*	*U*_iso_*/*U*_eq_	
I2	0.38469 (2)	0.37839 (12)	0.76874 (3)	0.0735 (2)	
C10	0.5931 (3)	0.3846 (12)	0.9095 (4)	0.0471 (15)	
H10A	0.6281	0.2874	0.9120	0.056*	
N2	0.6682 (3)	0.6372 (10)	1.0215 (4)	0.0513 (14)	
H2B	0.6678	0.7709	1.0513	0.062*	
H2C	0.6947	0.6550	0.9901	0.062*	
H2D	0.6823	0.5174	1.0599	0.062*	
C11	0.5297 (3)	0.3288 (13)	0.8534 (4)	0.0507 (16)	
H11A	0.5224	0.1926	0.8181	0.061*	
C9	0.6020 (3)	0.5850 (11)	0.9604 (4)	0.0412 (14)	
C8	0.5520 (3)	0.7336 (12)	0.9573 (4)	0.0503 (16)	
H8A	0.5596	0.8706	0.9923	0.060*	
C12	0.4790 (3)	0.4729 (12)	0.8504 (4)	0.0448 (15)	
C7	0.4894 (3)	0.6768 (13)	0.9008 (5)	0.0514 (16)	
H7A	0.4546	0.7769	0.8973	0.062*	
I1	0.11990 (2)	0.89729 (11)	0.38039 (3)	0.06947 (19)	
C3	−0.1001 (3)	1.0935 (11)	0.3605 (4)	0.0424 (14)	
N1	−0.1670 (2)	1.1490 (10)	0.3549 (4)	0.0528 (14)	
H1B	−0.1941	1.0391	0.3218	0.063*	
H1C	−0.1783	1.2916	0.3299	0.063*	
H1D	−0.1694	1.1499	0.4096	0.063*	
C6	0.0249 (3)	0.9855 (12)	0.3725 (4)	0.0451 (15)	
C2	−0.0884 (3)	0.8935 (12)	0.3196 (4)	0.0520 (16)	
H2A	−0.1228	0.7944	0.2883	0.062*	
C4	−0.0498 (3)	1.2412 (12)	0.4073 (4)	0.0497 (16)	
H4A	−0.0581	1.3774	0.4349	0.060*	
C5	0.0135 (3)	1.1858 (13)	0.4131 (5)	0.0543 (17)	
H5A	0.0479	1.2850	0.4445	0.065*	
C1	−0.0264 (3)	0.8386 (12)	0.3247 (4)	0.0511 (16)	
H1A	−0.0185	0.7033	0.2962	0.061*	
O6	0.3304 (2)	−0.1221 (9)	0.9162 (3)	0.0657 (14)	
N4	0.2778 (3)	−0.1278 (12)	0.8524 (4)	0.0553 (15)	
O5	0.2512 (3)	−0.3234 (10)	0.8268 (4)	0.0745 (16)	
O4	0.2531 (3)	0.0559 (11)	0.8148 (4)	0.0826 (18)	
N3	0.2248 (3)	0.3536 (11)	0.5730 (4)	0.0560 (15)	
O3	0.2487 (3)	0.1485 (9)	0.5783 (4)	0.0686 (15)	
O2	0.1727 (3)	0.3781 (10)	0.5883 (4)	0.0749 (16)	
O1	0.2517 (3)	0.5249 (11)	0.5535 (5)	0.0885 (19)	

### Atomic displacement parameters (Å^2^)

**Table:**

	*U*^11^	*U*^22^	*U*^33^	*U*^12^	*U*^13^	*U*^23^
I2	0.0463 (3)	0.1119 (5)	0.0590 (3)	−0.0160 (3)	0.0141 (2)	−0.0082 (3)
C10	0.044 (3)	0.054 (4)	0.045 (3)	0.005 (3)	0.017 (3)	−0.002 (3)
N2	0.048 (3)	0.054 (4)	0.053 (3)	−0.010 (3)	0.019 (3)	−0.005 (3)
C11	0.059 (4)	0.054 (4)	0.040 (3)	−0.003 (3)	0.019 (3)	−0.009 (3)
C9	0.038 (3)	0.048 (4)	0.038 (3)	−0.005 (3)	0.013 (3)	0.002 (3)
C8	0.060 (4)	0.042 (4)	0.051 (4)	−0.005 (3)	0.021 (3)	−0.005 (3)
C12	0.043 (3)	0.059 (4)	0.035 (3)	−0.004 (3)	0.017 (3)	0.002 (3)
C7	0.045 (4)	0.053 (4)	0.059 (4)	0.007 (3)	0.021 (3)	0.002 (3)
I1	0.0449 (3)	0.0962 (4)	0.0641 (3)	0.0188 (3)	0.0146 (2)	−0.0032 (3)
C3	0.043 (3)	0.047 (4)	0.040 (3)	0.003 (3)	0.017 (3)	0.000 (3)
N1	0.043 (3)	0.060 (4)	0.055 (3)	0.006 (3)	0.016 (3)	−0.003 (3)
C6	0.044 (3)	0.056 (4)	0.036 (3)	0.014 (3)	0.013 (3)	0.005 (3)
C2	0.051 (4)	0.056 (4)	0.049 (4)	−0.007 (3)	0.017 (3)	−0.012 (3)
C4	0.055 (4)	0.040 (4)	0.052 (4)	0.004 (3)	0.016 (3)	−0.009 (3)
C5	0.047 (4)	0.056 (4)	0.054 (4)	0.000 (3)	0.009 (3)	−0.014 (3)
C1	0.059 (4)	0.052 (4)	0.045 (4)	−0.001 (3)	0.021 (3)	−0.014 (3)
O6	0.058 (3)	0.069 (4)	0.056 (3)	0.000 (3)	0.002 (2)	−0.001 (3)
N4	0.044 (3)	0.061 (4)	0.061 (4)	−0.004 (3)	0.017 (3)	−0.005 (3)
O5	0.063 (3)	0.063 (3)	0.099 (4)	−0.019 (3)	0.030 (3)	−0.016 (3)
O4	0.069 (4)	0.069 (4)	0.085 (4)	0.000 (3)	−0.006 (3)	0.009 (3)
N3	0.046 (3)	0.058 (4)	0.062 (4)	0.005 (3)	0.016 (3)	−0.007 (3)
O3	0.061 (3)	0.057 (3)	0.095 (4)	0.015 (3)	0.037 (3)	−0.001 (3)
O2	0.055 (3)	0.071 (4)	0.114 (5)	0.008 (3)	0.048 (3)	−0.007 (3)
O1	0.070 (4)	0.063 (4)	0.143 (6)	0.001 (3)	0.050 (4)	0.010 (4)

### Geometric parameters (Å, °)

**Table:**

I2—C12	2.091 (6)	C3—N1	1.467 (7)
C10—C9	1.360 (9)	N1—H1B	0.8900
C10—C11	1.401 (9)	N1—H1C	0.8900
C10—H10A	0.9300	N1—H1D	0.8900
N2—C9	1.469 (7)	C6—C5	1.361 (9)
N2—H2B	0.8900	C6—C1	1.389 (9)
N2—H2C	0.8900	C2—C1	1.365 (9)
N2—H2D	0.8900	C2—H2A	0.9300
C11—C12	1.359 (9)	C4—C5	1.390 (9)
C11—H11A	0.9300	C4—H4A	0.9300
C9—C8	1.361 (9)	C5—H5A	0.9300
C8—C7	1.392 (9)	C1—H1A	0.9300
C8—H8A	0.9300	O6—N4	1.246 (7)
C12—C7	1.371 (9)	N4—O4	1.221 (8)
C7—H7A	0.9300	N4—O5	1.243 (7)
I1—C6	2.095 (6)	N3—O1	1.220 (8)
C3—C2	1.365 (9)	N3—O2	1.253 (7)
C3—C4	1.373 (9)	N3—O3	1.254 (7)
			
C9—C10—C11	118.2 (6)	C3—N1—H1B	109.5
C9—C10—H10A	120.9	C3—N1—H1C	109.5
C11—C10—H10A	120.9	H1B—N1—H1C	109.5
C9—N2—H2B	109.5	C3—N1—H1D	109.5
C9—N2—H2C	109.5	H1B—N1—H1D	109.5
H2B—N2—H2C	109.5	H1C—N1—H1D	109.5
C9—N2—H2D	109.5	C5—C6—C1	120.4 (6)
H2B—N2—H2D	109.5	C5—C6—I1	120.4 (5)
H2C—N2—H2D	109.5	C1—C6—I1	119.2 (5)
C12—C11—C10	120.3 (6)	C3—C2—C1	120.2 (6)
C12—C11—H11A	119.8	C3—C2—H2A	119.9
C10—C11—H11A	119.8	C1—C2—H2A	119.9
C10—C9—C8	122.4 (6)	C3—C4—C5	119.6 (6)
C10—C9—N2	117.7 (6)	C3—C4—H4A	120.2
C8—C9—N2	119.8 (6)	C5—C4—H4A	120.2
C9—C8—C7	118.8 (6)	C6—C5—C4	119.6 (6)
C9—C8—H8A	120.6	C6—C5—H5A	120.2
C7—C8—H8A	120.6	C4—C5—H5A	120.2
C11—C12—C7	120.5 (6)	C2—C1—C6	119.7 (6)
C11—C12—I2	119.2 (5)	C2—C1—H1A	120.1
C7—C12—I2	120.3 (5)	C6—C1—H1A	120.1
C12—C7—C8	119.8 (6)	O4—N4—O5	120.4 (6)
C12—C7—H7A	120.1	O4—N4—O6	120.4 (6)
C8—C7—H7A	120.1	O5—N4—O6	119.1 (7)
C2—C3—C4	120.6 (6)	O1—N3—O2	120.8 (6)
C2—C3—N1	119.4 (6)	O1—N3—O3	121.0 (6)
C4—C3—N1	120.0 (6)	O2—N3—O3	118.1 (6)

### Hydrogen-bond geometry (Å, °)

**Table:**

*D*—H···*A*	*D*—H	H···*A*	*D*···*A*	*D*—H···*A*
N1—H1B···O4^i^	0.89	2.18	2.945 (8)	144
N1—H1C···O2^ii^	0.89	2.24	2.819 (8)	122
N1—H1C···O5^i^	0.89	2.46	3.010 (8)	120
N1—H1D···O3^i^	0.89	2.46	2.898 (8)	111
N1—H1D···I1^ii^	0.89	3.15	3.990 (6)	157
N2—H2B···O6^iii^	0.89	2.03	2.892 (8)	162
N2—H2B···O4^iii^	0.89	2.44	3.104 (8)	132
N2—H2B···N4^iii^	0.89	2.55	3.372 (8)	153
N2—H2C···O3^iv^	0.89	1.91	2.795 (7)	173
N2—H2C···N3^iv^	0.89	2.56	3.407 (8)	159
N2—H2C···O1^iv^	0.89	2.59	3.264 (8)	133
N2—H2D···O5^v^	0.89	2.18	3.018 (8)	157
N2—H2D···O6^v^	0.89	2.28	3.053 (8)	145
N2—H2D···N4^v^	0.89	2.58	3.459 (9)	170

Symmetry codes: (i) −*x*, −*y*+1, −*z*+1; (ii) −*x*, −*y*+2, −*z*+1; (iii) −*x*+1, −*y*+1, −*z*+2; (iv) −*x*+1, *y*+1/2, −*z*+3/2; (v) −*x*+1, −*y*, −*z*+2.
